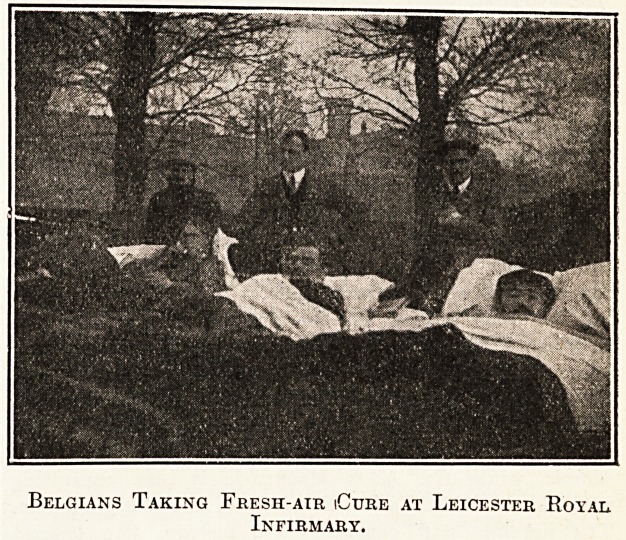# Hospitals and the War: Work for the Wounded of the Allied Armies

**Published:** 1914-12-12

**Authors:** 


### HOSPITALS AND THE WAR.

(From our Special Correspondents.)

### Work for the Wounded of the Allied Armies.

### CAMBRIDGE: MORE WOUNDED ARRIVE.

A further contingent of about 150 wounded soldiers arrived at Cambridge on November 25. There were only two "cot" cases—officers—and these were conveyed immediately by motor to the Research Hospital, Hills Road, Cambridge. The remaining 148 were conveyed in motor ambulances and cars, very kindly lent by Dr. Gray, Dr. Venn, Mr. Mellowes, Mrs. Peart, Mr. E. H. Parker, Mr. Lassiter, and others, to the 1st Eastern General Hospital.

A certain number were enabled to have refreshments, provided by members of Cambs No. 14 (Shelford) Voluntary Aid Detachment, under the direction of Commandant Mrs. Carter Jonas.

#### 250 Wounded Soldiers Arrive.

Last Saturday evening, December 5, about 250 wounded British soldiers from the northern part of France, where the battle has been raging fiercely, were brought from Southampton to Cambridge. They were received at Cambridge Station by Cambs No. 13 (Men's) Detachment of British Red Cross Society, under the command of Mr. Alger.

The wants of the wounded were attended to, and they were then removed in less than an hour to the 1st Eastern General Hospital.

### CARDIFF: WEATHER IN FRANCE.

Another ambulance train, conveying 123 wounded and sick British soldiers returning from the firing line, reached Cardiff on Monday afternoon (December 7) for treatment at the group of hospitals forming the 3rd Western General Hospital. Thirty-two of the cases were described as "cot," and the majority of those who were conveyed on stretchers by the members of the Glamorgan V.A.D. to the motor fleet in waiting were suffering from frozen feet. The cases were distributed as follows: Splott Road, 50; King Edward VII.'s Hospital, 15; Albany Road, 20; Howard Gardens, 38. Included among the soldiers were a number of Territorials belonging to London regiments, and a few members of the Royal Welsh Fusiliers. The men travelled from Boulogne on Sunday night, and said that they had encountered very severe weather during the fighting at Ypres. Snow, hail, and frost, they declared, had been a greater terror to them than the enemy's bullets.

### LEICESTER: 5th NORTHERN GENERAL HOSPITAL.

With the arrival of a further convoy of 180 wounded and sick soldiers from Flanders on December 5, the work of this hospital has become once again active after a period of comparative quiet. Full use has been made during the past few weeks of the facilities for treatment of convalescent patients in various convalescent homes and country houses in the county, with the result that the numerous transfers brought the number of available beds in the base hospital to nearly 300. There was thus ample accommodation for the new convoy, which arrived from Southampton at 4.30 last Saturday afternoon. Fifty were "cot" cases, many suffered acutely from frost-bite, but there were few wounds.

The men were mostly drawn from the Ypres region, but some had already been treated in the base hospitals in France, and their journey across the Channel was a very boisterous and trying one. It was, however, observed that they were all well provided with warm and suitable garments; as one of the Voluntary Aid Detachment nurses remarked, "They were beautifully clothed." The transport was, as on former occasions, in the hands of Mr. A. W. Faire, the County Director, and by 5.30 the last motor-car had arrived with its load at the hospital. Mr. Faire has been deservedly complimented on many occasions on his transport arrangements.

Belgians Taking Fresh-air Cure at Leicester Royal Infirmary.

The sick and wounded at the Leicester Royal Infirmary continue to make good progress. Our illustration shows a number of wounded Belgian soldiers taking the "fresh-air cure" in the grounds of the institution.

### WAKEFIELD: CLAYTON HOSPITAL.

Another batch of wounded soldiers arrived straight from the Front, after a very trying three days' journey from Boulogne, on November 19. The crossing of the Channel was a lengthy one, owing to the necessity for avoiding mines, etc., and when the men arrived by the Midland train at Leeds they were very tired. Motor-cars, kindly lent again by Wakefield friends, and two colliery ambulances from Altofts and Whitwood went to Leeds to fetch the wounded, and quickly got them back to the hospital at Wakefield. The injuries this time were not only wounds from shell, but also injuries from frost-bite and rheumatism, and one man among them had to be dug out of the trenches after being practically buried alive for two days and unconscious. The first batch of thirty-one have all now been discharged convalescent, and it is hoped that this new batch of thirty will also very soon get well.

One day last week General Menz and his staff paid a surprise visit to the hospital from York, and spent some time in the wards with the wounded soldiers, and on leaving expressed his entire satisfaction with the arrangements made and the way in which the wounded soldiers were being looked after.

### BRISTOL.

Bristol has again received further contingents of wounded; 129 arrived on November 30, among whom were men of the West Kent, Grenadier, Oxford Yeomanry, and Engineer regiments; and 150 came on December 2, the latter for the most part being cases due to exposure. Many of the men had been in hospital in England before transference to Bristol.

Saturday last was known locally as "Chocolate Saturday" (and Bristol among other things is a chocolate city), when an appeal was made by the Bristol branch of the Navy League for gifts of chocolate for the sailors and soldiers. A St. John Ambulance Association hospital has been opened at Newton Park, a wing of which building has been offered for the purpose by the Earl and Countess Temple, and already a number of patients have been received from the base hospital at Bristol. The Commandant of the hospital is Assistant Commissioner Sprawson, of the St. John Ambulance Brigade; the medical officers are Dr. Leslie Beath and Dr. Mary Morris, while Dr. Alick Mackenzie is hon. consulting physician and Mr. Forbes Fraser hon. consulting surgeon. The nursing staff consists of a matron, two trained nurses, four probationers, and two orderlies.

### DONCASTER: MANSION HOUSE FOR REFUGEES.

Since the early weeks of the war the Doncaster Voluntary Aid Detachment has maintained a hospital in the Mansion House, Doncaster, with fifty beds and a full supply of qualified Red Cross nurses. So far the expected patients have not put in an appearance, and owing to the room occupied by the hospital being required for municipal purposes, the Commandant, Mrs. Pickering, has arranged with the Mayor that all hospital beds and requisites shall, for the present, be packed in as small space as possible, and kept ready for immediate use when required. The hospital beds, however, have recently been the means of rescuing the Corporation from a position of some embarrassment. Arrangements were made by the Town Council, through a special committee, to receive and find accommodation for some fifty Belgian refugees in Doncaster. Two large houses were placed at the disposal of the committee, and it was intended to divide the refugees into two households of about twenty-five each. One of the houses was ready for immediate occupation, but the other was not, and it was arranged that the first party of twenty-five Belgians should be sent down from London on a Thursday, and the remainder should follow on the Monday ensuing. To the consternation of the committee almost the whole of the Belgians arrived *en masse*, with only two or three hours' notice. In this emergency the V.A.D. ladies at once offered the accommodation of their Mansion House hospital, and this tided the refugees over the week-end. Some time ago a large house known as Edenfield, Thorne Road, Doncaster, standing in its own grounds, was offered to the local V.A.D. as a convalescent home by its owner, Mr. Arnold. This palatial house has now been accepted by the War Office as a convalescent hospital for twenty-five soldiers, free of cost to the Government, for three months or longer. Meanwhile the ladies of the V.A.D. continue to maintain an officially recognised depôt in the Mansion House for clothing and other gifts intended for the troops.

### SHEFFIELD: A SOLDIER ON DOCTORS UNDER FIRE.

Until last week a considerable time had elapsed since the arrival of the last convoy of wounded in Sheffield. In consequence, a large number of the beds at the 3rd Northern General Hospital (Sheffield) were empty, and the staff, under Colonel Connell, M.D., were having a comparatively easy time. Of the men who had been in hospital, a large number had been sent to convalescent homes—that is, houses lent by various kindly people to the hospital authorities for the accommodation of wounded soldiers who were in a condition to leave the base hospital. Other men had been sent to their homes.

On Sunday night, November 29, however, a batch of 126 wounded British soldiers arrived at Sheffield from Ypres. Of these, twenty-five were "cot" cases, and there were a considerable number of instances of men who were suffering from severe frost-bite. Mr. F. M. Tindall organised the splendid motor-ambulance service.

Hardly had this batch of wounded men become accustomed to their new quarters than a second batch arrived. This second convoy consisted of 129 men who had been fighting in the neighbourhood of Ypres, and twenty-nine of the number required to be removed from the hospital train on stretchers. For the work of detraining Colonel Connell had the assistance of Major Dr. Pooley, Major Dr. Cuff, and a substantial detachment of men from the R.A.M.C.

Dr. Connell has received quite a number of letters from soldiers who have passed through the Sheffield Hospital, thanking him and the members of the medical and nursing staffs for the great kindness extended to them. Similar experiences are reported from St. John's Red Cross Hospital at Dore, and from the other institutions. Among other letters is one to a local medical man from a non-commissioned officer, now fighting with his regiment in France, who remarks, "I cannot express in writing what I feel towards the medical profession, both at home and abroad. You are all heroes. I have seen your brother officers dressing wounds under shell fire as coolly as if they were taking tea in their own homes."

## Figures and Tables

**Figure f1:**